# 2022 CMICS Expert Consensus on the Management of Isolated Tricuspid Regurgitation after Left-Sided Valve Surgery

**DOI:** 10.31083/j.rcm2405129

**Published:** 2023-04-26

**Authors:** Jinmiao Chen, Zhaoyun Cheng, Nianguo Dong, Lili Dong, Huiming Guo, Yingqiang Guo, Huanlei Huang, Shengli Jiang, Fanglin Lu, Fei Li, Jinping Liu, Liming Liu, Xin Li, Ju Mei, Liang Ma, Chenhui Qiao, Lizhong Sun, Guowei Tu, Liang Tao, Dongjin Wang, Huishan Wang, Minxin Wei, Song Wan, Jianjun Xu, Song Xue, Zhe Zheng, Lai Wei, Chunsheng Wang, on behalf of CMICS

**Affiliations:** ^1^Department of Cardiovascular Surgery, Zhongshan Hospital, Fudan University, 200032 Shanghai, China; ^2^Department of Cardiovascular Surgery, Fuwai Central China Cardiovascular Hospital, 451464 Zhengzhou, Henan, China; ^3^Department of Cardiovascular Surgery, Union Hospital, Tongji Medical College, Huazhong University of Science and Technology, 430022 Wuhan, Hubei, China; ^4^Department of Echocardiography, Zhongshan Hospital, Fudan University, 200032 Shanghai, China; ^5^Department of Cardiovascular Surgery, Guangdong Provincial People's Hospital, Guangdong Provincial Cardiovascular Institute, 510080 Guangzhou, Guangdong, China; ^6^Department of Cardiovascular Surgery, West China Hospital, Sichuan University, 610041 Chengdu, Sichuan, China; ^7^Department of Cardiovascular Surgery, Chinese PLA General Hospital, 100853 Beijing, China; ^8^Department of Cardiovascular Surgery, Changhai Hospital, Naval Medical University, 200433 Shanghai, China; ^9^Department of Cardiovascular Surgery, Zhongnan Hospital, Wuhan University, 430071 Wuhan, Hubei, China; ^10^Department of Cardiovascular Surgery, The Second Xiangya Hospital of Central South University, 410011 Changsha, Hunan, China; ^11^Department of Cardiothoracic Surgery, Xinhua Hospital, Shanghai Jiaotong University School of Medicine, 200092 Shanghai, China; ^12^Department of Cardiovascular Surgery, The First Affiliated Hospital, Zhejiang University School of Medicine, 310003 Hangzhou, Zhejiang, China; ^13^Department of Cardiovascular Surgery, The First Affiliated Hospital of Zhengzhou University, 450052 Zhengzhou, Henan, China; ^14^Department of Cardiovascular Surgery, Shanghai Deltaheath Hospital, 201702 Shanghai, China; ^15^Cardiac Intensive Care Center, Zhongshan Hospital, Fudan University, 200032 Shanghai, China; ^16^Department of Cardiovascular Surgery, Wuhan Asia Heart Hospital, 430022 Wuhan, Hubei, China; ^17^Department of Cardiothoracic and Vascular Surgery, Nanjing Drum Tower Hospital, Nanjing University, 210008 Nanjing, Jiangsu, China; ^18^Department of Cardiovascular Surgery, General Hospital of Northern Theater Command, 110016 Shenyang, Liaoning, China; ^19^Division of Cardiovascular Surgery, The University of Hong Kong-Shenzhen Hospital, 518000 Shenzhen, Guangdong, China; ^20^Division of Cardiothoracic Surgery, Prince of Wales Hospital, The Chinese University of Hong Kong, 999077 Hong Kong, China; ^21^Department of Cardiovascular Surgery, The Second Affiliated Hospital of Nanchang University, 330006 Nanchang, Jiangxi, China; ^22^Department of Cardiovascular Surgery, Renji Hospital, Shanghai Jiaotong University School of Medicine, 200127 Shanghai, China; ^23^Department of Cardiovascular Surgery, Fuwai Hospital, Chinese Academy of Medical Sciences and Peking Union Medical College, 100037 Beijing, China

**Keywords:** tricuspid regurgitation, left-sided valve surgery, surgical treatment, transcatheter therapy, intervention timing, expert consensus

## Abstract

Tricuspid regurgitation (TR) may occur late after left-sided valve surgery (LSVS). Isolated tricuspid regurgitation after left-sided valve surgery (iTR-LSVS) refers to isolated TR without significant lesions in the mitral and/or aortic position late after mitral and/or aortic replacement or repair. Severe TR has a negative impact on long-term prognosis and requires surgical or transcatheter treatment. However, there is no clear recommendation on when and how intervention should be performed for patients with iTR-LSVS in the current guidelines for the management of valvular heart disease. The historically high operative mortality may be reduced by current minimally invasive techniques and transcatheter therapy. To further understand iTR-LSVS, standardize the treatment, improve the prognosis, and promote the collaboration, the Chinese Minimally Invasive Cardiovascular Surgery Committee (CMICS) wrote this expert consensus on the management of iTR-LSVS from the aspects of etiology, preoperative evaluation, indications for intervention, surgical treatment, transcatheter therapy, and postoperative management.

## 1. Introduction

The continual development of cardiac surgery has benefited many patients with left-sided valve diseases. Some patients would have tricuspid regurgitation (TR) late after left-sided valve surgery (LSVS) during long-term follow-up. Isolated tricuspid regurgitation after left-sided valve surgery (iTR-LSVS) refers to isolated TR without significant lesions in the mitral and/or aortic position late after mitral and/or aortic replacement or repair. Severe TR can lead to right heart failure, liver and renal dysfunction, and systemic congestion and has a negative impact on long-term prognosis.

There is no clear recommendation on when and how intervention should be performed for patients with iTR-LSVS in the current guidelines for the management of valvular heart disease [1, 2, 3, 4]. The operative mortality of isolated tricuspid valve surgery (iTVS) was historically as high as 10% [5, 6, 7, 8, 9, 10, 11, 12, 13]. Minimally invasive techniques may reduce the operative mortality and complication rates despite lack of high-quality data [11, 12, 13, 14, 15, 16, 17, 18, 19]. Recently, transcatheter tricuspid valve repair or replacement is becoming an alternative for selected patients [20, 21]. To further understand iTR-LSVS, standardize the treatment, improve the prognosis, and promote the collaboration, the Chinese Minimally Invasive Cardiovascular Surgery Committee (CMICS) wrote this expert consensus.

## 2. Etiology

The tricuspid valve has been disregarded for a long time because TR is usually secondary to left-sided valve disease rather than primary lesion. It was widely believed that treatment of left-sided valve diseases would lead to spontaneous resolution of TR, but increasing evidence has shown that TR in some patients may remain or even worsen late after LSVS.

The pathogenesis of iTR-LSVS is complex and still not completely clear [22]. The potential underlying mechanisms include the following: (1) The effective orifice area of the left-sided prosthetic valve or ring is too small, which leads to relative valve stenosis, increased left atrial pressure, pulmonary hypertension, right ventricle (RV) dilation, and functional TR [23]. (2) Persistent atrial fibrillation causes dilation of the right atrium (RA) and tricuspid annulus [24]. (3) The rheumatic lesion involves tricuspid leaflets, which results in thickening, contracture and fusion of leaflets and organic TR [11]. (4) Mild or moderate TR is left untreated during prior LSVS or tricuspid repair without using annuloplasty rings [25]. (5) The pacemaker lead passes through the tricuspid valve and causes TR [26]. (6) Concomitant coronary artery disease may have a role in the development of TR [27].

Given these possible underlying mechanisms of late TR, there are some recommendations for LSVS: (1) Valve repair should be the first choice if possible. (2) Use prosthetic valve as large as possible. (3) Tricuspid valve repair should be considered in patients with mild or moderate TR and a dilated annulus during LSVS. (4) Concomitant surgical ablation is recommended in patients with atrial fibrillation.

## 3. Preoperative Evaluation

Transthoracic echocardiography is recommended to assess the severity of TR, the size and function of the RA and RV, and pulmonary artery pressure [28]. A new TR grading scheme is recommended (Table 1) [29, 30, 31, 32, 33]. The vena contracta width (VCW) should be measured from the parasternal four-chamber and parasternal RV inflow views and then averaged [34]. Velocity correction is recommended for two-dimensional proximal isovelocity surface area (PISA) measurement. The correction coefficient is $V_{\text{TRpeak}}$/($V_{\text{TRpeak}}$ – Va), in which Va is the aliasing velocity and $V_{\text{TRpeak}}$ is the peak velocity of TR [35]. The three-dimensional PISA method does not require velocity correction [36].

**Table 1.** **Classification of tricuspid regurgitation**.

Parameters	Mild	Moderate	Severe	Massive	Torrential
VCW (biplane)	<3 mm	3–6.9 mm	7–13 mm	14–20 mm	≥21 mm
EROA (PISA)	<20 ${\mathrm{mm}}^{2}$	20–39 ${\mathrm{mm}}^{2}$	40–59 ${\mathrm{mm}}^{2}$	60–79 ${\mathrm{mm}}^{2}$	≥80 ${\mathrm{mm}}^{2}$
3D VCA or quantitative ${\mathrm{EROA}}^{a}$	/	/	75–94 ${\mathrm{mm}}^{2}$	95–114 ${\mathrm{mm}}^{2}$	≥115 ${\mathrm{mm}}^{2}$

VCW, vena contracta width; EROA, effective regurgitant orifice area; PISA, proximal isovelocity surface area; 3D VCA, three-dimensional vena contracta area. ^a^3D VCA and quantitative Doppler EROA cut-offs may be larger than PISA EROA.

The systolic function of the RV in the longitudinal direction usually decreases after cardiac surgery with compensation by the radial direction [37]. Accurate evaluation of the RV systolic function with any single index is difficult [38, 39]. Therefore, measurement of both tricuspid annular plane systolic excursion (TAPSE) and right ventricular fractional area change (FAC) is preferred. When TAPSE is less than 16 mm and/or FAC is less than 35%, systolic dysfunction of the RV is indicated.

Right heart catheterization is a critical method for evaluating RV function, which can accurately measure RA and RV pressure, pulmonary artery pressure, pulmonary capillary wedge pressure, cardiac output and cardiac index. It is recommended to perform right heart catheterization preoperatively if available, especially for patients with poor RV function and severe pulmonary artery hypertension. Cardiac output, cardiac index and RV diastolic pressure reflect RV function. Pulmonary vascular resistance reflects RV afterload. Pulmonary capillary wedge pressure indicates left atrial pressure. It is worth noting that there is certain discordance in pulmonary artery pressure estimated by echocardiography and right heart catheterization in severe TR, as the former tends to underestimate or overestimate pulmonary artery pressure [40]. Cardiac magnetic resonance imaging may be helpful for measurement of RV volume and function. Tests of liver and renal function, coagulation function and complete blood count are also necessary.

When pulmonary capillary wedge pressure is higher than 15 mmHg, the function of the left-sided valves should be assessed. It is recommended to consider the necessity of concomitant surgery for the left-sided valves based on the patient’s status if the following conditions occur. In the mitral position, (1) the effective orifice area is less than 2 ${\mathrm{cm}}^{2}$ due to pannus on the prosthetic valve or mobility disorders of the prosthetic leaflets or (2) moderate or severe mitral regurgitation or paravalvular leak exist. In the aortic position, severe prosthetic valve stenosis or regurgitation exists.

Preoperative risk stratification is critical for patient selection. The Society of Thoracic Surgeons (STS) score and the EuroSCORE II are not suitable for iTVS. A few models are dedicated to iTVS, such as the Clinical Risk Score, TRI-SCORE, and MELD Score [41, 42, 43]. However, patients with TR are highly heterogeneous, and different etiologies determine different prognoses [5, 11, 12, 44, 45]. iTR-LSVS is more common in China than in Western countries and has a relatively poor prognosis among different kinds of TR [46]. The value of the abovementioned models for iTR-LSVS is still unclear. A thorough preoperative assessment is necessary for each patient, and a specific risk scoring model for iTR-LSVS should be built [47, 48].

## 4. Indications for Intervention

Based on recent studies, intervention is recommended for patients with the following conditions.

(1) Severe TR.

(2) Normal left ventricular function with ejection fraction ≥50%.

(3) Symptoms of right heart failure, such as ascites, peripheral edema, pleural effusions, and poor appetite, or asymptomatic or mild symptoms but progressive right heart dilatation and dysfunction.

Any following conditions should be considered high-risk.

(1) Severe RV dysfunction, that is, TAPSE less than 10 mm or FAC less than 25%.

(2) Pulmonary artery systolic pressure greater than 60 mmHg or pulmonary vascular resistance greater than 4 wood units.

(3) Liver cirrhosis or elevated blood ammonia.

The timing of intervention is an important determinant of postoperative outcome. The current guidelines have no clear recommendation due to the lack of evidence [1, 2, 3, 4]. One reason for the historically high mortality of iTVS after LSVS may be the delay in referral to surgery, which may lead to irreversible RV failure [5, 6, 7, 8, 9, 10]. Thus, it is gradually accepted that early surgical correction should be performed before the development of irreversible RV dysfunction and end-organ damage, despite a lack of high-quality randomized controlled trials [49].

## 5. Surgical Treatment

**Repair or replacement**: The severity of leaflet pathological lesions and tricuspid annulus dilation, right heart function, and the feasibility of repair should be taken into consideration when choosing the therapeutic strategy. Tricuspid valve repair (TVr) is preferred for patients with mild leaflet pathological lesions, and those who are expected to have favorable outcomes with reliable repair techniques. TVr techniques generally include annuloplasty with a prosthetic ring, leaflet patch augmentation, edge-to-edge, artificial chordae implantation, and other techniques [13]. In contrast, tricuspid valve replacement (TVR) is likely to be suitable for patients with significant rheumatic damage in leaflets, those with severe leaflet tethering due to severe dilation of the RV and tricuspid annulus, and those who are likely to have uncertain outcomes even after complex repair techniques [12, 50]. Long-term follow-up data are still required to compare the outcomes of TVr and TVR for patients with iTR-LSVS.

**Choice of prosthetic valve**: For TVR, the selection of prosthetic valve is still controversial [51]. The relevant meta-analyses showed that there are no significant differences between mechanical and bioprosthetic valves in terms of survival or reoperation rate after TVR [52, 53, 54, 55]. Considering that mechanical valves in the tricuspid position have a higher risk of valve thrombosis and that patients with iTR-LSVS are usually elderly and fragile, bioprosthetic valves may be preferred when patients are aged >60 years [12, 56]. However, there is no clear recommendation about the age at which it is reasonable to use a bioprosthetic valve in the tricuspid position in the current American College of Cardiology/American Heart Association (ACC/AHA) and European Society of Cardiology/European Association for Cardio-Thoracic Surgery (ESC/EACTS) guidelines [1, 2]. With the development of the tricuspid valve-in-valve technique, bioprosthetic valves may be used in younger patients in the tricuspid position, but more data are still needed [57, 58].

**Surgical approach**: For patients without obvious adhesions in the right thoracic cavity, it might be suitable for endoscopy-assisted or totally endoscopic surgery via a right minithoracotomy in the 4th intercostal space. A temporary pacemaker lead is recommended to be placed through the internal jugular vein preoperatively. The arterial cannula is inserted through the femoral artery with the Seldinger technique. The venous cannula is inserted through the femoral vein to the superior vena cava without dissection or ligation of the vena cava. The beating-heart technique is used without clamping the ascending aorta. Right atriotomy is performed directly through the pericardium without liberation of the RA. Vacuum-assisted femoral venous drainage, maintaining less than –40 mmHg negative pressure, can suck up most of the blood and ensure a clean operative view. The surgical field is filled with carbon dioxide at a flow of 0.5–1.0 L/min. For patients with obvious adhesions in the right thoracic cavity, median thoracotomy can be used. For patients who need concomitant left-sided valve reoperation, the surgical approach should be determined based on a comprehensive preoperative evaluation.

## 6. Transcatheter Therapy

Various transcatheter tricuspid valve repair and replacement systems are undergoing preclinical or clinical trials, and their early safety and feasibility have been confirmed. The current transcatheter tricuspid valve repair devices include TriClip (Abbott Vascular, Santa Clara, CA, USA), PASCAL (Edwards Lifesciencies, Irvine, CA, USA), FORMA (Edwards Lifesciencies, Irvine, CA, USA), TriAlign (Mitralign, Tewksbury, MA, USA), Cardioband (Edwards Lifesciencies, Irvine, CA, USA), K-Clip (Huihe Medical Technology, Shanghai, China) and others [59, 60, 61, 62, 63, 64]. The available transcatheter tricuspid valve replacement systems include NaviGate (NaviGate Cardiac Structures, Lake Forest, CA, USA), LuX-Valve (Jenscare Biotechnology, Ningbo, China), Evoque (Edwards Lifesciencies, Irvine, CA, USA) and others [20, 65, 66].

Despite convincing evidences in the field of percutaneous treatment of TR are relatively limited, transcatheter therapy may provide additional benefits over medical therapy alone [67, 68]. Recently, a randomized controlled trial demonstrated that transcatheter edge-to-edge repair was safe for patients with symptomatic severe tricuspid regurgitation, and the severity of tricuspid regurgitation was reduced to moderate or less at 1 year in the majority of patients [69]. Although these studies did not focus on iTR-LSVS patients and long-term outcomes were lack, transcatheter therapy can be considered for iTR-LSVS patients with clinical symptoms, prohibitive or high risk for surgical treatment, and suitable anatomical conditions [70].

## 7. Postoperative Management

One of the high-risk periods for RV failure is the early postoperative phase. The increased postoperative RV afterload, as well as perioperative hypoxemia, hypercapnia and acidosis, may aggravate RV failure. The clinical manifestations of RV failure include systemic hypotension, organ dysfunction, and elevated RV filling pressure. Patients might also suffer from acidosis and decreased mixed venous oxygen saturation, while the increases in pulmonary artery pressure and pulmonary capillary wedge pressure depend on the severity of left ventricular dysfunction or preoperative pulmonary hypertension.

Determining the fluid responsiveness of the patient is the key to manage RV failure. For patients with cardiogenic shock and fluid overload, renal replacement therapy should be started early for “negative fluid resuscitation” [71]. Norepinephrine or vasopressin is the first option to maintain blood pressure in patients with hypotension. Dobutamine or milrinone can also reduce RV afterload by dilating the pulmonary artery [72]. Factors that might induce pulmonary hypertension should be avoided, and low-tidal-volume mechanical ventilation should be given insofar as oxygenation and ventilation conditions allow. Inhalation of nitric oxide is another effective therapy to reduce pulmonary vascular resistance for RV failure when conventional therapies are not effective [73, 74]. Prostaglandin analogs, such as iloprost and prostacyclin, can also be adopted as adjunct treatments for intractable RV failure.

## 8. Conclusions

TR worsens the long-term prognosis of patients with iTR-LSVS. The timing and methods of intervention are of utmost significance. It is generally accepted that intervention should be performed before irreversible RV failure occurs. For patients with mild tricuspid valve lesions and patients who are expected to achieve favorable outcomes with reliable repair techniques, TVr is recommended. Otherwise, TVR may be a better choice. Minimally invasive techniques may reduce the operative mortality and complication rates. The evolving transcatheter tricuspid valve repair or replacement will become an alternative for selected patients with iTR-LSVS in the future. This expert consensus reflects the current state of the art and is designed to evolve with the field.
